# Practical Departments

**Published:** 1905-11-11

**Authors:** 


### PRACTICAL DEPARTMENTS.

### "VELURE," A JAPAN PAINT FOR HOSPITALS AND SIMILAR INSTITUTIONS, PUBLIC BUILDINGS, etc.

"Velure" is what is termed a Japan paint. It is intended to take the place of the ordinary lead paint, both for inside and outside work, at less cost it is claimed, and with greater convenience. It should be of great value for hospitals, as it is applicable to brick, stone, plaster, iron, wood, or practically any substance, and to old walls as well as new ones. Its appearance when properly applied is similar to that of enamel, and it is stated to have been used for inside walls, in place of glazed tiles. The surface of the wall, when painted with "velure," keeps remarkably clean. It does not get soiled and dirty as soon as the ordinary paint, and when it is dirty can easily be cleaned by washing with either hot or cold water, the cleansing rendering it as fresh as when first put on, so that for the walls of hospital wards and corridors it should be a great boon. For old walls that have been already painted, it is recommended that the old paint should be completely removed before the "Velure" is applied, and for this purpose another substance appropriately called "Stripso" is provided, that will remove any kind of paint, by simply destroying it, the "Stripso" being laid on over the old paint, allowed to remain for a little time, and the whole removed together.

As an instance of its efficacy a case is given where a tradesman's shop counter had received three coats of Japan black, four of enamel, and four of ordinary paint, "Stripso" was applied on Saturday afternoon, and the whole of the paint removed on Monday morning with a brush and cold water, leaving the wood quite clean. "Velure" is also claimed to be a good damp preventive, when applied to walls exposed to moist prevailing winds. In short, "Velure" appears to be a great boon to everyone who has a wall or a ceiling to protect, either inside or outside. It is very easily applied, if the instructions sent out with it are carefully followed.

